# The cytokine IL-27 reduces inflammation and protects photoreceptors in a mouse model of retinal degeneration

**DOI:** 10.1186/s12974-022-02576-x

**Published:** 2022-09-05

**Authors:** Andrea Nortey, Kimberly Garces, Tal Carmy-Bennun, Abigail S. Hackam

**Affiliations:** Bascom Palmer Eye Institute, University of Miami Miller School of Medicine, Miami, FL 33136 USA

**Keywords:** Cytokine, Photoreceptors, IL-27, Retinal degeneration, Inflammation

## Abstract

**Background:**

Retinal degenerative diseases are a group of conditions characterized by photoreceptor death and vision loss. Excessive inflammation and microglial activation contribute to the pathology of retinal degenerations and a major focus in the field is identifying more effective anti-inflammatory therapeutic strategies that promote photoreceptor survival. A major challenge to developing anti-inflammatory treatments is to selectively suppress detrimental inflammation while maintaining beneficial inflammatory responses. We recently demonstrated that endogenous levels of the IL-27 cytokine were upregulated in association with an experimental treatment that increased photoreceptor survival. IL-27 is a pleiotropic cytokine that regulates tissue reactions to infection, neuronal disease and tumors by inducing anti-apoptotic and anti-inflammatory genes and suppressing pro-inflammatory genes. IL-27 is neuroprotective in the brain, but its function during retinal degeneration has not been investigated. In this study, we investigated the effect of IL-27 in the *rd10* mouse model of inherited photoreceptor degeneration.

**Methods:**

Male and female *rd10* mice were randomly divided into experimental (IL-27) and control (saline) groups and intravitreally injected at age post-natal day (P) 18. Retina function was analyzed by electroretinograms (ERGs), visual acuity by optomotor assay, photoreceptor death by TdT-mediated dUTP nick-end labeling (TUNEL) assay, microglia/macrophage were detected by immunodetection of IBA1 and inflammatory mediators by cytoplex and QPCR analysis. The distribution of IL-27 in the retina was determined by immunohistochemistry on retina cross-sections and primary Muller glia cultures.

**Results:**

We demonstrate that recombinant IL-27 decreased photoreceptor death, increased retinal function and reduced inflammation in the *rd10* mouse model of retinal degeneration. Furthermore, IL-27 injections led to lower levels of the pro-inflammatory proteins Ccl22, IL-18 and IL-12. IL-27 expression was localized to Muller glia and IL-27 receptors to microglia, which are key cell types that regulate photoreceptor survival.

**Conclusion:**

Our results identify for the first time anti-inflammatory and neuroprotective activities of IL-27 in a genetic model of retinal degeneration. These findings provide new insight into the therapeutic potential of anti-inflammatory cytokines as a treatment for degenerative diseases of the retina.

## Background

Retinal degenerations are characterized by dysfunction and death of light-sensing rod and cone photoreceptors, leading to vision loss and eventual blindness. As photoreceptors degenerate, they release molecules that stimulate inflammatory signaling and recruit microglia to the outer nuclear layer (ONL) [1–5]. Microglia act in concert with macrophages, glia and other inflammatory cells to regulate the overall neuroinflammatory response. Early inflammatory reactions to CNS damage are believed to be reparative, but prolonged injury in situations such as chronic disease or mutation causes excessive inflammatory activation and results in neuronal toxicity [6–9]. During retinal degeneration, reactive microglia and infiltrating macrophages induce phagocytosis of healthy photoreceptors and contribute to the progression of retinal damage [10–12]. Mouse knock-outs for key inflammatory genes indicate that excessive inflammation accelerates retinal degeneration, whereas inhibiting microglial-mediated inflammation reduces photoreceptor death in multiple mouse models of retinal degeneration [13, 14]. Furthermore, experimental therapies that induce photoreceptor survival in animal models often lead to reduced microglia activation, indicating the close relationship between retinal homeostasis and inflammation [5]. However, broad-acting anti-inflammatory drugs have limited efficacy and protective effects in the retina are transient, in part due to these drugs reducing reparative and anti-inflammatory signals in addition to blocking pro-inflammatory neurotoxic signals [6–8, 13]. Therefore, targeted strategies that dampen pro-inflammatory signaling and maintain reparative inflammatory pathways would be more beneficial to injured neurons than eliminating all inflammation. The objective of this study is to identify a mechanism of controlling excessive inflammation in order to promote photoreceptor survival.

IL-27 is an anti-inflammatory and cytoprotective heterodimeric cytokine in the IL-12 superfamily. IL-27 suppresses inflammation and promotes neuronal survival in rodent models of multiple sclerosis, uveitis, stroke and intracerebral hemorrhage [15–19]. Furthermore, IL-27 may also contribute to GM-CSF-induced survival of dopaminergic neurons [20] and matrine-induced neuroprotection in the EAE mouse model [18]. We recently demonstrated that higher endogenous IL-27 levels were associated with photoreceptor protection induced by an innate immunity inhibitor in the *rd10* mouse model of inherited retinal degeneration [21], suggesting a potential role for IL-27 in the degenerating retina.

IL-27 is a major downstream target gene of multiple inflammatory pathways and is induced by TLRs, IFN, CD40 and other pathways [19]. IL-27 comprises the Epstein–Barr virus induced gene 3 (EBI3) and IL-27p28, and engages a heterodimeric receptor composed of gp130 and IL-27Rα, which activates Janus kinase (JAK)-signal transducer and activator of transcription (STAT) and mitogen activated protein kinase (MAPK) signaling. EBI3 and IL-27p28 are induced in antigen-presenting cells and multiple glial and neuronal cell subtypes [19, 22] and IL-27 and its receptors are rapidly increased after neuronal damage [22, 23]. IL-27Rα is specific for IL-27 and is expressed on the cell surface of neurons, glia, microglia and other inflammatory cells.

Recent studies described dichotomic functions of IL-27 as pro- or anti-inflammatory depending on the tissue and cell types (reviewed in [24]). In many tissues, IL-27 signals through the JAK–STAT pathway and induces anti-apoptotic genes Bcl-2 and Bcl-XL and suppresses pro-inflammatory cytokines such as TNFα, IL-1β and iNOS. IL-27 also increases expression of immunoregulatory cytokine IL-10 and endogenous NF-κB inhibitors and reduces NLRP3 inflammasome activation [25, 26]. In contrast, IL-27 is pro-inflammatory in tumors by regulating T cell effector functions [27]. Previous work in the retina demonstrated that IL-27 is upregulated in photoreceptors and microglia in a mouse uveitis model and IL-27 leads to STAT1-dependent induction of IL-10 and SOCS1, suppressing intraocular inflammation, inhibiting expansion of Th17 cells, and preserving the retina [22, 28]. However, whether IL-27 regulates inflammation and photoreceptor survival in a genetic model of retinal degeneration, which does not show T cell involvement, has not been investigated.

In this study, we investigated whether IL-27 has anti-inflammatory and neuroprotective activities in the retina. Our findings demonstrate that IL-27 increased photoreceptor survival, stabilized visual acuity and rescued rod and cone functional responses in the *rd10* retinal degeneration model. Photoreceptor protection was associated with lower levels of inflammation. Furthermore, IL-27 was detected in Muller glia and IL-27 receptors are expressed in microglia. These findings are the first demonstration of a protective effect for IL-27 during an inherited neurodegeneration and suggest that IL-27 has potential for further development as a novel anti-inflammatory therapeutic strategy.

## Methods

### Procedures with animals

All experiments involving mice were approved by the Animal Care and Use Committee at the University of Miami and were carried out in accordance with the ARVO statement for the Use of Animals in Ophthalmic and Vision research. The retinal degeneration 10 *(rd10)* mouse model was purchased from Jackson Laboratory and housed with a 12-h light–dark cycle, free access to food and water, and all cages were maintained at similar distances from the overhead lights. Experimental and control treatments were from the same litters.

Mice were divided randomly into experimental groups and were injected intravitreously with sterile saline (control) or IL-27 (10, 20, 40 ng/µl) (BioLegend, San Diego, CA) on post-natal day (P) 18, as follows. The mice were anesthetized using isoflurane, the corneas were anesthetized with a drop of 0.5% proparacaine hydrochloride and the mice were placed under a surgical microscope on a heated pad to maintain body temperature at 37 °C. The eyes were intravitreally injected with 1 µl of solution using a 33-gauge Hamilton needle (Hamilton Company, Reno, NV) passed through the sclera into the vitreous cavity and angled to avoid the lens. The fellow eye was uninjected. Mice with bleeding or swelling were excluded from further study. Investigators were masked to the treatment for all subsequent analyses.

### Electroretinogram (ERG)

Scotopic and photopic ERGs were performed using the UTAS system and EM for Windows software (LC Technologies, Gaithersburg, MD), as described previously [29]. Seven days after injection of IL-27 or saline, the mice were anesthetized with a ketamine/xylazine mixture, pupils were dilated with 2.5% phenylephrine hydrochloride and body temperature was maintained using a Physitemp controller. A reference electrode was inserted subcutaneously on the forehead, corneal silver wire electrodes were placed on both eyes, and a ground electrode was inserted at the base of the tail then the mice were placed into a Ganzfield light-emitting chamber. For recording rod photoreceptor-driven responses, dark adapted mice were exposed to flashes of white light at intensities − 1 to + 1 log cd s/m^2^. Similarly, cone-driven photopic responses to flashes of green light were recorded after a brief period of light adaptation. The responses to 10 flashes of light with an interstimulus time of 5 s were recorded and averaged for each light intensity then a-wave and b-wave amplitudes were derived from the ERG waves.

### Visual acuity testing

Reflexive optomotor tracking movements were used to evaluate vision using an optomotor system in which the spatial frequency, contrast and speed of the stimulus is controlled by an OptoMotry-AT device (CerebralMechanics, Inc.), following the method of [30] and as described previously [31]. Two investigators who were masked to the treatment observed whether the animal tracked the movement of rotating black and white columns. Visual acuity thresholds were determined using the staircase procedure in which the thickness of the columns decreased step-wise until the mouse was no longer able to track the columns. Visual acuity was defined as the highest spatial frequency that generated a consistent head-tracking response. Asymmetric tracking, in which the tracking response is higher in one direction, reflects different acuities of the two eyes and can be used to assess the response to treatment [32].

### Immunohistochemistry

Mice were humanely euthanized by CO_2_/cervical dislocation at P25 and the eyes were immediately removed for processing. The eyes were fixed in 4% paraformaldehyde for 1 h then incubated overnight in increasing sucrose concentrations from 5 to 20% and then embedded in Optimal Cutting Temperature Compound (OCT) (Sakura, Tissue-Tek) and frozen. The entire eye was sectioned into 10-μm cryostat-cut sections and mounted onto glass slides. Slides from equivalent regions of the eye among treatment groups were used. The slides were washed in PBS, incubated in blocking buffer (10% goat serum in 0.3% Triton X-100/PBS) for 30 min then incubated overnight at 4 °C in primary antibody solution containing anti-Iba1 antibody (Wako, Richmond, VA, 1:200 dilution), anti-IL-27p28 antibody (Novus, Centennial, CO, 1:100 dilution) or anti-GFAP (Invitrogen, 1:400 dilution), diluted in 2% goat serum in 0.3% Triton X-100/PBS. The slides were then washed in PBS and incubated in Alexa Fluor secondary antibody (Invitrogen, 1:600 dilution) for 1 h at room temperature. Control slides were incubated with only secondary antibody to confirm lack of non-specific immunostaining. The slides were counterstained with 4′6-diamidino-2-phenylindole (DAPI) in Vectashield mounting media and imaged with a Zeiss fluorescent microscope. Exposure times were equivalent among the control slides and all experimental groups. The microscope images were then coded by an independent investigator to mask the treatment. Microglia/macrophage counts were performed by counting Iba1-positive cells in nine non-adjacent retinal sections per animal and cell density was calculated by dividing the number of cells by the length of the region counted.

Primary Muller glia cultures were prepared from C57BL/6 J mouse pups age P7-8 following the procedures described in [33]. The cultures were passaged onto glass coverslips, fixed in 4% paraformaldehyde and immunostained with anti-IL-27p28 antibody or anti-GFAP, as above. The purity of the Muller glia cultures was confirmed by morphology and by immunodetection with anti-glutamine synthase antibody (Abcam, Cambridge, MA; 1:1000).

### Quantification of photoreceptor death

To detect dying cells, ten micron cryostat retina sections were processed using a TdT-mediated dUTP nick-end labeling (TUNEL) assay according to the manufacturer directions (Promega, Madison, WI, USA). Briefly, sections were incubated with proteinase K, reacted with TdT/nucleotide mix containing fluorescein-12-dUTP and counterstained with DAPI then the slides were viewed with a Zeiss fluorescent microscope at 20 × magnification. Negative controls were used to confirm specificity. The total number of TUNEL-positive/DAPI-positive nuclei in the ONL was counted along the entire retina section in at least six non-adjacent sections per retina.

### Quantification of inflammatory proteins

Levels of inflammatory proteins were analyzed using a LEGENDplex Mouse Inflammation Panel kit (BioLegend, San Diego, CA). Mice were injected intravitreously with 20 ng IL-27 or saline at P18 and retinas were collected at P21 and lysed in PBS containing protease inhibitors and 0.1% NP40. Cytokine assays were performed on the samples according to the manufacturer’s directions and protein levels in samples and standard curves were detected using a CytoFlex S apparatus (Beckman Coulter) then analyzed with LEGENDplex analysis software.

### Quantitative PCR (QPCR) and standard PCR

Retinas from injected mice were collected at P25, total RNA was extracted using TRIZOL reagent (Invitrogen) then 1 µg of RNA was reverse transcribed into cDNA using SuperScript IV (Invitrogen). QPCR amplification on cDNA was performed in triplicate for each sample using 2X Universal SYBR Green Fast qPCR Mix (Abclonal, Woburn, MA) in a Realplex2 Mastercycler (Eppendorf) using primers specific to the genes of interest that spanned at least one intron: *Nos2* forward 5′-CAGAGGACCCAGAGACAAGC-3′, *Nos2* reverse 5′-TGCTGAAACATTTCCTGTGC-3′, *Trem2* forward 5′-CAGCCCTGTCCCAAGCCCTCAAC-3′, *Trem2* reverse 5′-CTCCTCACCCAGCTGCCGACACC-3′. The delta-delta Ct method was used for relative gene quantification using the housekeeping gene ARP as the reference gene [34], with the calculations in https://toptipbio.com/delta-delta-ct-pcr. Additionally, standard PCR was performed on cDNA prepared from RNA extracted from whole wild-type C57Bl/6 J mouse retina, cultured BV-2 microglia cells [35] or primary Muller glia cultures [33] using Taq polymerase (NEB). The primers used were: *IL-27Rα* forward 5′-CTCCTGGGAACCTTTGGGC-3′, *IL-27Rα* reverse 5′-GGCCGTCCCTTTTGTGTCCCCC-3′, *IL-27* forward TGTCCACAGCTTTGCTGAAT-3′, *IL-27* reverse 5′-AAGGGCCGAAGTGTGGTAG-3′. Control reactions that lacked the cDNA template were included to confirm absence of contamination.

### Statistical analysis

Statistical analysis was performed using GraphPad Prism. Statistical differences among experimental groups were determined using ANOVA followed by Tukey’s multiple comparisons tests or two-tailed unpaired Student’s *t*-test. *p* values < 0.05 were considered statistically significant and data are reported as mean ± standard deviation unless otherwise specified.

## Results

The *rd10* mouse strain is a commonly used inherited photoreceptor degeneration that mimics human retinitis pigmentosa in which rod and cone photoreceptors die at a predictable rate due to a mutation in the rod-specific PDE6β gene [36]. Onset of rod photoreceptor death in *rd10* mice typically begins during the third week of life and is followed by secondary cone degeneration [37, 38]. We recently reported that an inhibitor of innate immunity increased photoreceptor survival in *rd10* and was associated with reduced inflammation and increased levels of IL-27 and other proteins [21, 39]. Furthermore, IL-27 has been shown to reduce inflammation and protect neurons in various mouse models of neuronal disease [15–19]. Therefore, to investigate whether IL-27 dampens inflammation in the degenerating retina and promotes photoreceptor survival, we intravitreally injected recombinant IL-27 into *rd10* mice early in degeneration at P18 and analyzed the effect on inflammation, photoreceptor survival and retinal function.

**Fig. 1:**
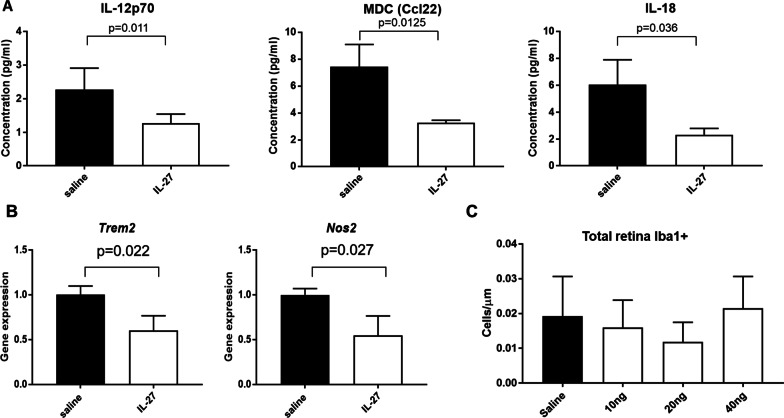
IL-27 reduced pro-inflammatory markers. **a** Inflammatory protein analysis. Significantly lower levels of IL-18, Ccl22 and IL-12p70 proteins in retinas from mice injected with 20 ng IL-27 compared with saline in retinas obtained 3 days after injection (*n* = 4). **b** QPCR analysis demonstrated that intravitreal injections of 20 ng IL-27 reduced pro-inflammatory microglia markers *Nos2* and *Trem2* compared to saline injections in retinas obtained 7 days after injection at P25 (*n* = 3). **c** Quantification of Iba1 + microglia/macrophage cells in the outer nuclear layer demonstrated similar levels among treatments (*n* = 3)

To assess whether IL-27 regulates inflammation in *rd10* mice, we measured the effect of IL-27 on several different inflammatory markers. First, we analyzed levels of cytokines and other inflammatory proteins in the retinas obtained three days after injection of IL-27 or saline, at a time-point prior to photoreceptor death. This analysis demonstrated significant reductions in levels of pro-inflammatory proteins IL-18, IL-12p70 and Ccl22 in retinas injected with IL-27 (Fig. 1A). In contrast, several cytokines in the panel that were consistently detected above background did not show significant reductions in the IL-27 injected retinas, including TNFα, IL-1β, GCSF, and IL-23.

To determine whether these early changes in inflammatory proteins were associated with changes in cellular responses at later time points, *rd10* mice were injected with 20 ng IL-27 or saline and QPCR analysis was performed on retinas obtained 7 days after injection. IL-27 reduced levels of pro-inflammatory microglia markers *Nos2* and *Trem2* compared to saline injections (Fig. 1B), suggesting lower levels of activated microglia and macrophages. However, IL-27 did not lead to a statistically significant decline in total numbers of Iba1-positive microglia/macrophage in the retina compared with saline-injected mice at any of the doses tested (Fig. 1C). Together, these data suggest that inflammatory molecules were reduced by IL-27 but the total number of inflammatory cells did not change.

**Fig. 2:**
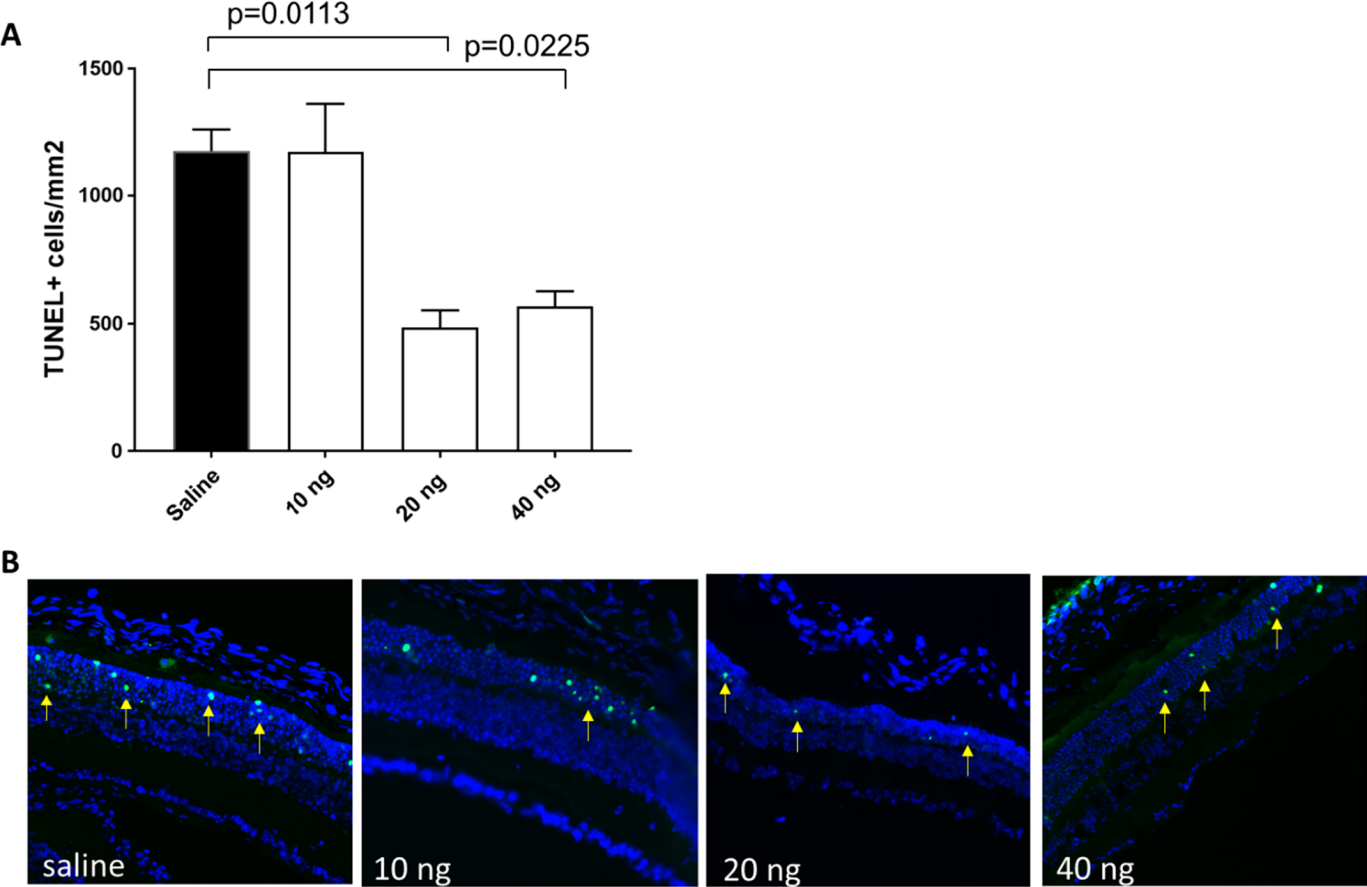
IL-27 reduced photoreceptor death. **a** Quantification of TUNEL-positive cells in retina cross-sections in *rd10* at P25 indicates lower photoreceptor death in the IL-27 injected mice compared with saline (*n* = 3). **b** Representative images showing green TUNEL-positive cells (yellow arrows) in the ONL of treated mice

We next tested whether IL-27 altered the extent of photoreceptor death. Retinas from saline or IL-27-injected mice were analyzed using TUNEL to quantify dying photoreceptors. The number of TUNEL-positive cells in the ONL was 976 cells/mm^2^ in the saline control injections (Fig. 2A). In contrast, significantly fewer TUNEL-positive photoreceptors were detected in the 20 ng (486 cells/mm^2^) and 40 ng IL-27 (569 cells/mm^2^) injected eyes, whereas the 10 ng dose had equivalent numbers as saline. Therefore, IL-27 reduced photoreceptor degeneration in *rd10* mice at a dosage of 20 ng and higher.

**Fig. 3:**
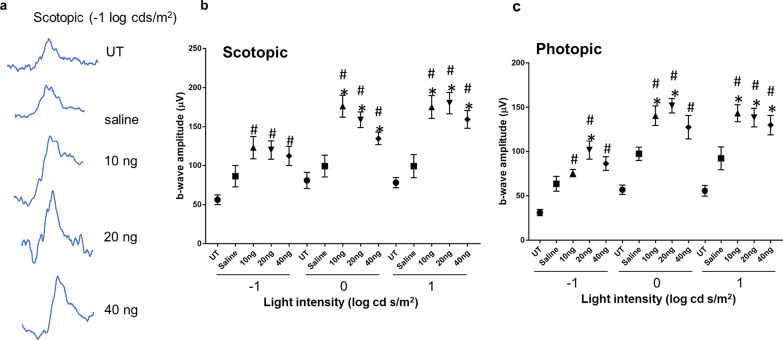
IL-27 leads to higher retinal function.** a** Representative ERG waves for photopic stimulation at flash intensity − 1 log cd s/m^2^. **b** Scotopic and **c** photopic b-wave amplitudes are shown for the IL-27 injected *rd10* mice compared with untreated and saline-injected mice using ERG recordings measured at age P25. Responses to three different flash intensities are shown. IL-27 increased b-wave amplitudes compared with saline and untreated mice. Statistical significance of *p* < 0.05 is marked with an asterisk (*) for IL-27 vs saline comparisons and a number sign (#) for IL-27 vs untreated comparisons. The average values for untreated mice are shown as circles (*n* = 10), saline injected as squares (*n* = 8), 10 ng IL-27 injected as triangles (*n* = 9), 20 ng IL-27 as inverted triangles (*n* = 11), and 40 ng IL-27 as diamonds (*n* = 12). Averages and standard error are shown

To determine whether reduced photoreceptor death led to increased retina function, ERGs were used to measure the activity of rod and cone photoreceptors in response to flashes of light at specific light intensities and wavelengths. ERG b-wave amplitudes represent the activity of inner retinal neurons that are stimulated by photoreceptors and correspond to photoreceptor function and survival [29]. Mice injected with IL-27 showed significantly higher rod (scotopic) (Fig. 3A) and cone (photopic) (Fig. 3B) responses compared with saline-injected controls at all light intensities. Furthermore, compared to untreated *rd10* mice [21], significantly higher ERG responses were measured in the IL-27 injected mice that were up to threefold greater than untreated mice (Fig. 3B). The 10 ng dose also showed elevated ERG responses indicating that this low dose was sufficient to increase function although the TUNEL data above showed that it was not sufficient to reduce death. The saline-injected mice were equivalent to untreated mice at most light intensities. Although the 20 ng dose resulted in higher responses than the other doses for most of the flash intensities, the difference among doses did not reach statistical significance. ERG a-waves were low as expected for *rd10* at this timepoint but were significantly higher in mice injected with IL-27 compared with saline for the highest two scotopic intensities (data not shown). Therefore, the ERG results demonstrate that IL-27 increased retinal function in a mouse model of severe retinal degeneration.

**Fig. 4:**
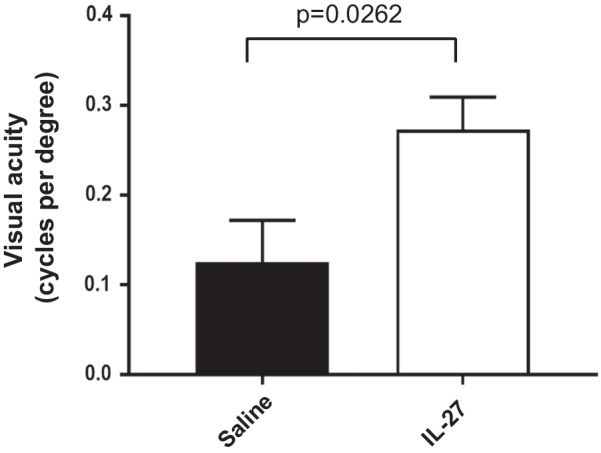
IL-27 improves visual acuity. *rd10* mice injected with IL-27 had significantly higher visual acuity than saline-injected mice. Mice were analyzed at age P32. *N* = 8 (IL-27) and 9 (saline), *p* = 0.0262

Next, we investigated whether improved ERGs and lower TUNEL counts early in degeneration would be associated with improved vision at a later timepoint. The optomotor reflex was used to quantify visual acuity. In wild-type mice, visual acuity reaches maximum at P25 then is stable throughout adulthood [30] whereas in *rd10* mice reduced visual acuity is evident by P27 and declines rapidly [40]. Visual acuity was measured in the IL-27 and saline-injected mice at P25 then again at P33 to determine whether vision declined or was stable. The IL-27-injected mice had equivalent acuity at both time points with only 6.3% reduction and no significant difference between P25 and P33. In contrast, saline-injected mice showed a substantial decline (46.4%) between the timepoints (*p* = 0.035, comparing P25 and P33). The difference in vision reduction between IL-27 and saline was significant (*p* = 0.025). Furthermore, visual acuity at age P33 was significantly higher in the IL-27 treated mice (Fig. 4), indicating that IL-27 stabilized visual acuity in the *rd10* mouse. Therefore, the ERG, TUNEL and optomotor data together demonstrate that IL-27 promoted photoreceptor survival.

**Fig. 5:**
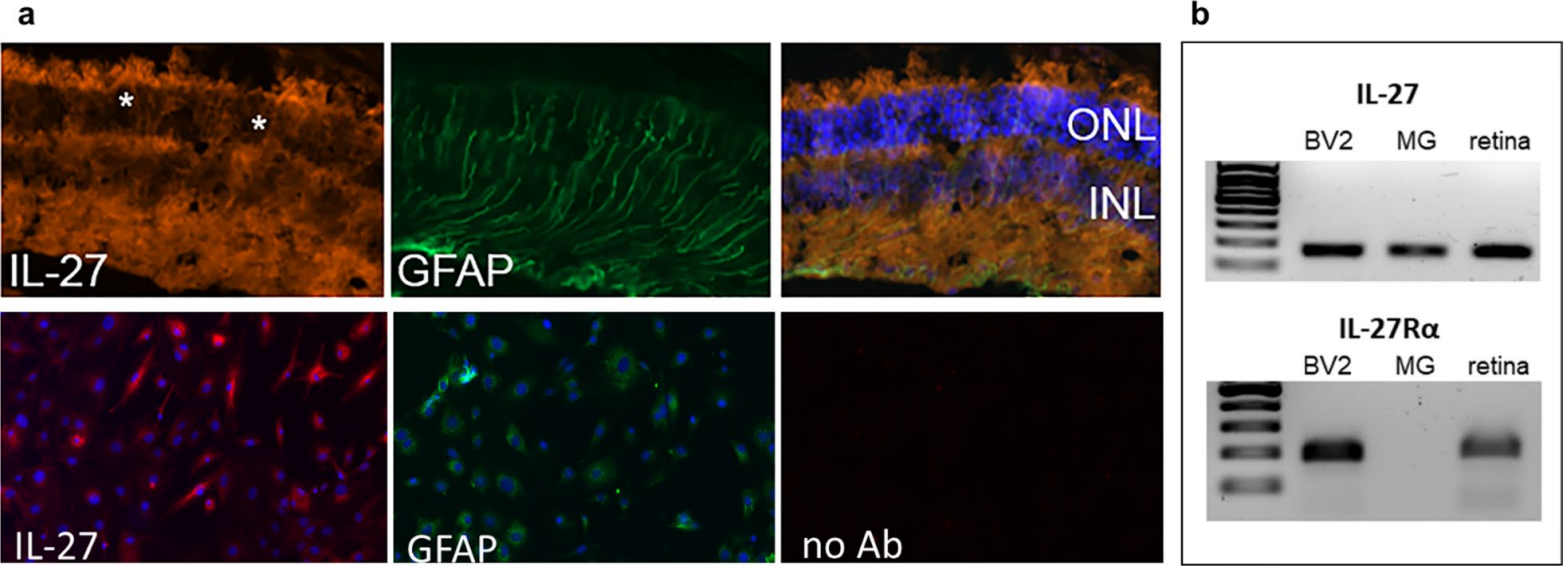
IL-27 localization in the retina. **a** (Upper panels) Immunodetection of IL-27p28 in *rd10* mice at age P25. Colocalization of IL-27p28 signal (red) with GFAP-positive processes (green) indicated by asterisks, consistent with expression in Muller glia. Brightness was enhanced in the GFAP panel in order to visualize the processes in the ONL. (Lower panels) Immunodetection of IL-27p28 in primary Muller glia cultures (red). GFAP (green) detection on an adjacent coverslip indicates the purity of the cultures because every cell was GFAP-positive. The “no Ab” panel contains primary Muller glia with no primary antibody at the equivalent exposure settings as the IL-27 image to demonstrate lack of non-specific immunodetection. GCL, ganglion cell layer; INL, inner nuclear layer; ONL, outer nuclear layer. **b** DNA gel with PCR products showing IL-27 and IL-27Rα amplification in the BV2 mouse microglia cell line, primary Muller glia (MG) and whole retina from *rd10* mice

Endogenous IL-27 is primarily secreted from inflammatory cells such as microglia or dendritic cells and its receptors are expressed in a variety of cell types, including neurons, astrocytes, macrophage and microglia (reviewed in [24]). To investigate which cell types might contribute to anti-inflammatory and neuroprotective effects of IL-27 in the retina, IHC and PCR were used on whole retina and isolated cells. Immunodetection using an antibody against the IL-27p28 subunit demonstrated expression in the ganglion cell layer (GCL), inner plexiform layer (IPL), outer plexiform layer (OPL) and inner segment region (IS) (Fig. 5A). This immunodetection pattern was confirmed on sections from multiple animals, was absent in the no primary antibody negative control, and is consistent with the localization reported by Lee et al. [22]. The immunostaining pattern demonstrated expression of endogenous IL-27 in Muller glia, which have processes that extend from the GCL to the ONL. IL-27p28 was co-detected with GFAP-positive processes indicating that IL-27 is expressed in Muller glia (Fig. 5A), which was confirmed by strong expression of IL-27 in cultured mouse primary Muller glia by IHC (Fig. 5A) and PCR (Fig. 5B). Unfortunately, immunodetection of the IL-27Rα receptor subunit using multiple antibodies was inconclusive. However, PCR analysis demonstrated that IL-27Rα is expressed in whole retina and in the BV-2 mouse microglia cell line but was not expressed in primary mouse Muller glia (Fig. 5B). Although BV-2 are not derived from retinas, they have many properties of retinal and CNS microglia. Therefore, endogenous IL-27 and its receptor are expressed in *rd10* retina and are localized to cell types that are important for regulating photoreceptor viability.

## Discussion

Precise control of neuroinflammatory signals is essential for restoring tissue homeostasis after CNS injury. Insufficient inflammation is predicted to reduce protective immune responses that remove dying neurons and limit damage, whereas excessive inflammation would recruit inflammatory cells leading to further tissue damage and increased neuronal death [6–9]. The purpose of the current study was to characterize the effect of exogenous IL-27 under conditions of elevated neurotoxic inflammation during retinal degeneration. Our findings indicate that IL-27 reduced inflammatory signals and improved photoreceptor survival in a mouse model of rapid photoreceptor death. This study is the first to identify survival and anti-inflammatory effects of IL-27 during retinal degeneration and adds new information about regulatory signals that control inflammation in the retina.

The protective effect of IL-27 in the retina adds to a growing body of evidence supporting pro-survival activities of IL-27 in the CNS [24]. Recent studies demonstrated that IL-27 reduced ischemia–reperfusion injury in the brain in a mouse model [15] and decreased neurological deficits and brain pathology in a model of intracerebral hemorrhage [17]. IL-27 also suppresses intraocular inflammation and protects the retina in a mouse uveitis model [22]. Additionally, an IL-27-neutralizing antibody reduced endogenous IL-27 and enhanced neurological deficits after intracerebral hemorrhage [17]. IL-27 is induced by other pro-survival molecules and potentially contributes to their cytoprotective functions, including GM-CSF-induced survival of dopaminergic neurons in a mouse Parkinson’s disease model [20], and matrine-dependent neuroprotection in the EAE mouse model [18].

Inflammation contributes to degeneration in *rd10* mice by stimulating microglia and promoting their migration to the ONL where they phagocytose healthy as well as dying photoreceptors [11]. The observation that IL-27 led to lower levels of the chemotactic cytokine Ccl22, which stimulates monocytes [41, 42], pro-inflammatory cytokine IL-12p70, which stimulates cytotoxic microglial responses [43], and pro-inflammatory cytokine IL-18, suggests that IL-27 protects photoreceptors by suppressing microglial activation in the photoreceptor layer. These reduced cytokines were detected 3 days post-injection and prior to photoreceptor death, suggesting that reduced inflammation led to lower levels of subsequent photoreceptor death. IL-27 also reduced expression of activated microglia markers Trem2 and Nos2 at later timepoints, but additional studies will be needed to confirm that lower Ccl22 and IL-12p70 led to decreased microglia activation. We also cannot rule out the possibility that reduced Trem2 and Nos2 was a consequence and not a cause of lower photoreceptor death.

Ccl22 is produced by microglia and leukocytes and is a chemoattractant for monocytes, dendritic cells and other cell types during inflammatory conditions [44]. Ccl22 and its receptor Ccr4 are associated with inflammation and pathogenesis in a mouse model of MS [45, 46]. Interestingly, Ccl22 is often used as a marker gene for reparative M2-type macrophage cells because these macrophages secrete Ccl22 to attract Th2 cells that promote fibrosis and wound healing [44]. However, the role of Ccl22 during retinal degeneration, which does not involve T lymphocytes, is unknown, but it is likely involved in enhancing pathological inflammation. Indeed, multiple studies have demonstrated upregulated Ccl22 during retinal inflammatory conditions, including retinal detachment [47], ischemic retinal vein occlusion [48] and neovascular AMD [49]. Inflammatory cytokines such as IL-1β, TNF and TLR activators induce Ccl22 secretion from macrophages and dendritic cells [50, 51]. Similar to the findings in the current study, lower Ccl22 levels and decreased macrophage infiltration were associated with therapeutic effects from reducing S100 calcium binding protein B in an animal model of retinal inflammatory disease called autoimmune uveoretinitis [41]. Zhang et al. also demonstrated that decreased Ccl22 and lower infiltration of macrophages and other inflammatory cells was associated with reduced brain injury in a hypoxic ischemic model from delivery of the CSF1R inhibitor PLX3397 [52]. Future experiments will determine whether suppressing Ccl22-dependent recruitment of microglia and macrophages contributes to IL-27 induced photoreceptor survival.

The observation of reduced IL-12p70 in IL-27-injected *rd10* retinas are consistent with previous studies on neuroprotective molecules in which blocking microglial activation and promoting neuronal survival were associated with lower IL-12 expression in various animal models of retina [53] and brain injuries [54]. IL-12p70 is a heterodimeric cytokine composed of p35 and p40 subunits and is in the same cytokine family as IL-27. IL-12 is often used as a marker of M1-type macrophages and is produced by macrophages and microglia, including retinal microglia [55], and activates T cells and NK cells, induces IFNγ secretion and stimulates cytotoxic responses [56]. Similar to Ccl22, IL-12 was elevated in animal models of retinal degeneration, including light toxicity and diabetic retinopathy [57, 58]. Although the roles of IL-12 in stimulating T cell responses and blocking pathological angiogenesis are well-described, the function of IL-12 in the retina has not been characterized. Future studies will extend our findings to investigate whether IL-12 contributes to neurotoxic inflammation and whether depleting IL-12 dampens inflammation and protects photoreceptors.

Another possible mechanism of neuroprotection by IL-27 could be activation of anti-apoptotic pathways in photoreceptors. IL-27 was reported to reduce apoptosis by acting directly on cultured neurons [15] and IL-27 stimulates expression of anti-apoptotic genes Bcl-2 and Bcl-XL [16, 59]. However, we did not observe differential expression of anti-apoptotic genes Bcl-XL, Survivin or c-myc in IL-27 injected retinas using QPCR analysis (data not shown). Therefore, our findings are most consistent with an anti-inflammatory effect of IL-27, which indirectly leads to photoreceptor protection by decreasing secretion of neuroinflammatory proteins. The reduced expression of multiple inflammatory cytokines by IL-27 may tip the balance to promoting an immunosuppressive and neuroprotective tissue environment. Additionally, Amadi-Obi et al. [60] demonstrated that IL-27 induces complement factor H (CFH) expression in a human RPE cell line ARPE-19 and in isolated retina cells from the mouse, suggesting that IL-27 regulates the complement cascade. Complement activation has been shown in *rd10* mouse retinas and knocking out C3 and its receptor decreased microglial-mediated removal of dying photoreceptors, which was associated with increased photoreceptor degeneration [61]. Therefore, IL-27 may function through a CFH-C3-C3R mechanism to regulate microglial activity and photoreceptor death. Future experiments, for example with RNAseq for detailed molecular profiling, will investigate underlying molecular changes contributing to IL-27 induced neuroprotection.

IL-27 signaling is well-described in T cells and dendritic cells, which are cell types not typically found in *rd10* retinas. Microglia, macrophages and other APC also secrete IL-27 and express IL-27 receptors [22, 62] and these cell types compose a major portion of the neuroinflammatory response to CNS injury. IL-27 may contribute to fine-tuning their coordinated response to damage in order to limit further neuronal death [24]. It is notable that IL-27 signaling components were detected in Muller glia and microglia, which are key cell types that influence photoreceptor survival. Muller glia express IL-27 (Fig. 5) and retinal microglia express both IL-27Rα and IL-27 [28]. It is possible that Muller glia may contribute to local inflammatory responses by secreting endogenous IL-27 that amplifies IL-27 signaling in microglia. Support for multiple glial types regulating an IL-27 signaling network is from a study by Lee et al. showing that IL-27 produced by retinal neurons and glia both contribute to suppressing intraocular inflammation and preserving the retina in a mouse uveitis model [22]. Future studies will determine the precise contribution of Muller glia to IL-27 signaling and will determine how cytokines signal between glial types to influence inflammation, and ultimately, photoreceptor survival. Studies will also investigate how IL-27 is regulated in the retina, whether it acts directly on photoreceptors and immune cells, and whether it is protective in other retinal disease models.

## Conclusion

We demonstrated using histological, molecular, functional and behavioral assays that a single intravitreal injection of the cytokine IL-27 induced significant photoreceptor protection and suppressed inflammation in a mouse model of retinal degeneration. Increased photoreceptor survival was associated with reduced markers of neuroinflammation and lower levels of IL-18, IL-12p70 and Ccl22. Therefore, these data indicate that IL-27 has potential for further development as a novel therapeutic strategy for retinal degenerations.

## Data Availability

The datasets obtained in the current study are available from the corresponding author on reasonable request.

## Abbreviations

ERG: Electroretinogram
TUNEL: TdT-mediated dUTP nick-end labeling
IL-27: Interleukin-27
ONL: Outer nuclear layer
P: Post-natal day
